# Dynamic Integrative Immune Profiling Reveals Early Biomarkers of Response and Prognosis in Advanced Gastric Cancer Treated with Nivolumab Plus Chemotherapy

**DOI:** 10.3390/cancers17193131

**Published:** 2025-09-26

**Authors:** Hyunho Kim, Kabsoo Shin, Se Jun Park, Myung Ah Lee, Juyeon Park, Okran Kim, Nahyeon Kang, In-Ho Kim

**Affiliations:** 1 Division of Medical Oncology, Department of Internal Medicine, St. Vincent Hospital, College of Medicine, The Catholic University of Korea, Seoul 06591, Republic of Korea; h2kim@catholic.ac.kr; 2 Division of Medical Oncology, Department of Internal Medicine, Seoul St. Mary’s Hospital, College of Medicine, The Catholic University of Korea, Seoul 06591, Republic of Korea; kabsoo.shin@catholic.ac.kr (K.S.); psj6936@naver.com (S.J.P.); angelamd@catholic.ac.kr (M.A.L.); pke1001@hanmail.net (J.P.); okrane@hanmail.net (O.K.); bluesky8473@catholic.ac.kr (N.K.); 3 Cancer Research Institute, College of Medicine, The Catholic University of Korea, Seoul 06591, Republic of Korea

**Keywords:** advanced gastric cancer, nivolumab, immune profiling, early biomarker, cytotoxic T cells, immune checkpoints, memory T cells

## Abstract

**Background**: Nivolumab plus chemotherapy is a standard first-line treatment for advanced gastric cancer (GC), but reliable early biomarkers for predicting treatment outcomes remain lacking. This study aimed to identify early immunological predictors through dynamic immune profiling. **Methods**: Fifty patients with advanced or unresectable GC receiving nivolumab plus XELOX or FOLFOX were enrolled. Peripheral blood was collected at baseline, week 1, and week 6. Plasma biomarkers (Granzyme B, Ki-67, CXCL10, IFN-γ, TGF-β1) were measured by ELISA, and immune cell subsets, including cytotoxic T cells, immune checkpoint–positive populations, and memory T-cell subsets, were analyzed by flow cytometry. Cutoffs were defined by medians, established thresholds for NLR and lymphocyte count, and criteria for long-term response (≥9.5 months). Associations with response and progression-free survival (PFS) were evaluated using Kaplan–Meier analysis, Cox regression, and ROC curves. **Results**: Early responders exhibited significant increases in Granzyme B and CXCL10, with ΔGranzyme B alone and in combination with ΔKi-67 predicting response with high accuracy. A lower week 1 neutrophil-to-lymphocyte ratio was associated with long-term benefit. Elevated week 1 CD8^+^ T-cell proportion and greater decreases in PD1^+^CD69^+^Ki-67^+^CD8^+^ T cells were linked to improved PFS. Higher baseline PD1^+^LAG-3^+^Ki-67^+^CD8^+^ T-cell levels and combined TIM-3^+^/LAG-3^+^ expression enhanced prognostic stratification. Additionally, elevated baseline activated TEMRA cells and declines at week 6 in the same subset correlated with better outcomes. **Conclusions**: These findings highlight the clinical utility of serial immune monitoring to enable early treatment stratification and guide personalized immunotherapy strategies in advanced GC.

## Simple Summary

Advanced gastric cancer has limited treatment options and poor prognosis. Nivolumab plus chemotherapy offers clinical benefit, but predictive biomarkers remain unclear. We analyzed blood samples from patients before and after treatment to assess immune activation markers, including cytotoxic molecules and CD8^+^ T cell subsets. Early increases in plasma Granzyme B and CXCL10, and specific activated CD8^+^ T cells, were associated with better outcomes. Some immune cell subsets showed marked declines after chemotherapy, indicating both stimulatory and suppressive immune effects. These findings may help identify patients most likely to benefit from immunotherapy, supporting personalized treatment strategies in advanced gastric cancer.

## 1. Introduction

Advanced gastric cancer (GC) remains a leading cause of cancer-related mortality worldwide, with poor long-term survival despite recent advances in systemic therapy [1]. Since the landmark CheckMate 649 trial, the combination of nivolumab with platinum-fluoropyrimidine chemotherapy has become a widely adopted first-line regimen for patients with advanced GC [2]. Beyond antibody-based checkpoint inhibitors, peptide-based antagonists are emerging as novel targeted therapeutics with potential applications in cancer immunotherapy [3]. In current clinical practice, a programmed death-ligand 1 (PD-L1) combined positive score (CPS) of ≥5 serves as the primary biomarker for patient selection [2,4]. In addition, microsatellite instability-high (MSI-H) status and Epstein–Barr virus (EBV) positivity have emerged as promising biomarkers that may guide patient selection for immunotherapy, reflecting the distinct immune microenvironment of these subgroups [5]. However, beyond these markers, no validated biomarkers are available to further refine patient stratification or optimize treatment decisions.

Importantly, immune responses during chemo-immunotherapy are highly dynamic, reflecting not only baseline immune status but also treatment-induced changes in immune activation, exhaustion, and memory formation [6,7,8,9]. Real-time monitoring of these changes may provide critical insights into treatment efficacy, enable early identification of responders and non-responders, and inform the development of novel therapeutic strategies [10].

In this context, the present study aimed to comprehensively characterize early and longitudinal changes in plasma immune markers and peripheral immune cell subsets in patients with advanced GC receiving nivolumab plus chemotherapy. By correlating these immune dynamics with treatment response and progression-free survival (PFS), we sought to identify novel prognostic and predictive biomarkers that could complement PD-L1 CPS and improve clinical decision-making.

## 2. Materials and Methods

### 2.1. Patient Population and Study Design

This observational study included 50 patients with metastatic or unresectable advanced gastric cancer who received nivolumab in combination with chemotherapy at Seoul St. Mary’s Hospital between July 2021 and September 2023. Integrative immune profiling was performed, incorporating analyses of cytotoxicity markers, immune checkpoint expression, and memory T-cell subsets to capture the dynamic immune landscape during treatment.

Eligible patients had histologically confirmed adenocarcinoma or poorly cohesive carcinoma, received at least two cycles of treatment, and underwent radiologic response assessment. Patients with HER2-positive tumors or prior exposure to immune checkpoint inhibitors were excluded. Radiologic evaluation was performed using contrast-enhanced computed tomography (CT) and assessed according to the Response Evaluation Criteria in Solid Tumors (RECIST) version 1.1. Baseline demographic and clinical data were collected from medical records.

The study was approved by the Institutional Review Board of Seoul St. Mary’s Hospital (KC18TNSI0361), and written informed consent was obtained from all patients.

### 2.2. Treatment Regimen

Nivolumab was administered at a fixed dose of 360 mg every 3 weeks or 240 mg every 2 weeks, in combination with chemotherapy. Chemotherapy consisted of either capecitabine plus oxaliplatin every 3 weeks or leucovorin, fluorouracil, and oxaliplatin every 2 weeks, according to institutional protocols and physician discretion.

### 2.3. Blood Sampling and Immune Monitoring

Peripheral blood was collected at three timepoints: pre-treatment (within 1 day before or on the day of treatment initiation), week 1 after treatment start, and week 6 (at the time of the first radiologic response assessment). The week 6 sampling was performed only in patients who maintained consent and for whom sample collection was feasible, resulting in a smaller subset for this timepoint analysis.

Peripheral blood mononuclear cells (PBMCs) were isolated using Ficoll–Hypaque density-gradient centrifugation. Flow cytometry (FACS) was performed to quantify immune cell subsets, including CD8^+^CD3^+^ T cells, PD1^+^CD8^+^ T cells, PD1^+^CD69^+^Ki67^+^CD8^+^ T cells, PD1^+^CD69^+^GranzymeB^+^CD8^+^ T cells, and CD69^+^TEMRA (terminally differentiated effector memory CD45RA^+^) cells, using commercially available antibody panels (BioLegend, San Diego, CA, USA, Cat# 300910, 310912, 329908, 151212, 372208, 304112, 369206, 345014; Thermo Fisher Scientific, Waltham, MA, USA, Cat# L34965). Plasma levels of Granzyme B, Ki-67, CXCL10, IFN-γ, and TGFβ1 were measured using commercially available enzyme-linked immunosorbent assay (ELISA) kits (BioLegend, Cat# 151212, 372208). All experiments were performed according to the manufacturer’s instructions.

### 2.4. Definition of Groups and Cutoff Values

Treatment response was categorized as complete response (CR) or partial response (PR) for responders, and stable disease (SD) or progressive disease (PD) for non-responders.

Cutoff values were defined as follows: for immune cell subset analysis, the median value was used; for the neutrophil-to-lymphocyte ratio (NLR), a threshold of 3.5 was applied based on prior literature in gastric cancer; and for the absolute lymphocyte count, 1500/μL was adopted, consistent with commonly accepted normal values (WBC 4000–5000/μL with about 30% lymphocytes). Long-term responders were defined as those with a duration of response ≥ 9.5 months, according to the median duration reported for the nivolumab plus chemotherapy group in the CheckMate 649 trial. For dynamic changes, Δ (delta) values were calculated as week 1 minus baseline levels.

### 2.5. Statistical Analysis

Progression-free survival (PFS) and overall survival (OS) were estimated using the Kaplan–Meier method, and differences between groups were evaluated with the log-rank test. Hazard ratios (HRs) and 95% confidence intervals (CIs) were calculated using Cox proportional hazards regression models. Receiver operating characteristic (ROC) curve analysis was performed to assess the predictive performance of biomarkers for treatment response, with the area under the curve (AUC) reported. Pairwise correlations between continuous immune marker values were assessed using Pearson’s correlation coefficient.

All statistical analyses were performed using R software (version 4.3.1; R Foundation for Statistical Computing, Vienna, Austria). Two-sided *p*-values < 0.05 were considered statistically significant.

## 3. Results

### 3.1. Participants Characteristics

A total of 50 patients with advanced gastric cancer were included in this study (Table 1). Histologically, adenocarcinoma was the most common type (*n* = 32), followed by poorly cohesive carcinoma (*n* = 18). Peritoneal carcinomatosis was present in 19 patients, liver metastasis in 9 patients, and bone metastasis in 5 patients. No patients were HER2-positive, whereas 7 patients had microsatellite instability-high (MSI-H) tumors and 4 were Epstein–Barr virus (EBV) positive. PD-L1 CPS ≥ 5 was observed in 28 patients. Forty-two patients received nivolumab in combination with XELOX, and the remaining 8 patients received nivolumab with FOLFOX. Among all 50 patients, the best overall response was an objective response in 28 patients.

### 3.2. Early Immune Response and Dynamic Changes in Cytotoxicity

**Figure 1:**
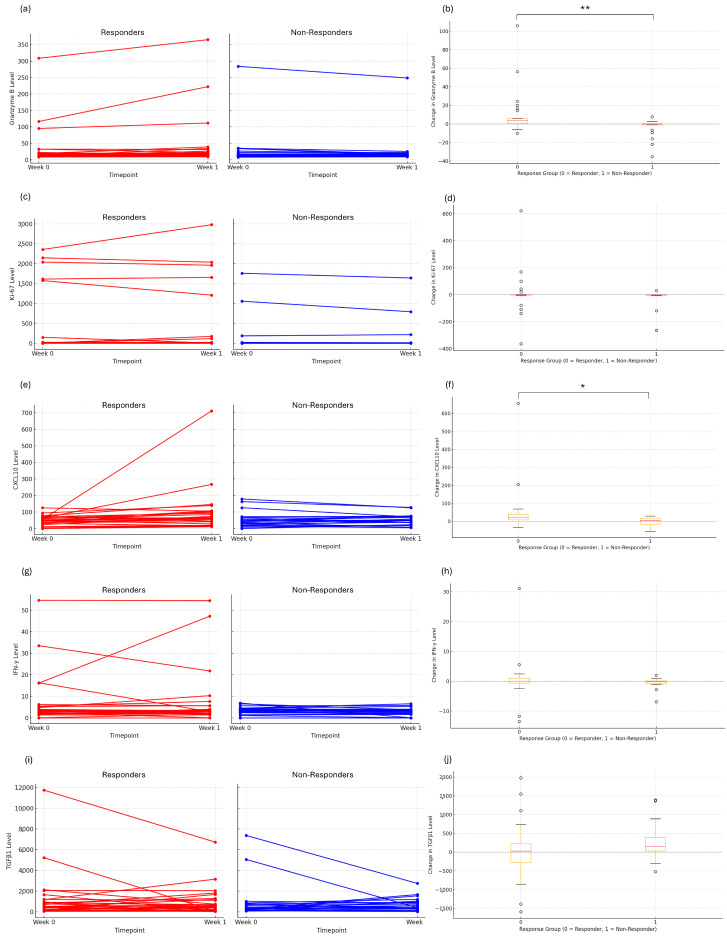
Early immune response and cytotoxic activation markers in patients with advanced gastric cancer receiving nivolumab plus chemotherapy. (**a**) Changes in plasma Granzyme B from baseline to week 1 in responders and non-responders; (**b**) ΔGranzymeB (week 1—baseline) according to response group; (**c**) Changes in Ki67 levels from baseline to week 1; (**d**) ΔKi67 (week 1—baseline) according to response group; (**e**) Changes in CXCL10 levels from baseline to week 1; (**f**) ΔCXCL10 (week 1—baseline) according to response group; (**g**) Changes in IFNγ levels from baseline to week 1; (**h**) ΔIFNγ (week 1—baseline) according to response group; (**i**) Changes in TGFβ1 levels from baseline to week 1; (**j**) ΔTGFβ1 (week 1—baseline) according to response group. Error bars indicate the standard error of the mean (SEM). Statistical significance was determined using the Mann–Whitney U test (* *p* < 0.05; ** *p* < 0.01).

Patients with CR or PR were classified as responders, whereas those with SD or PD were classified as non-responders. Plasma levels of Granzyme B, Ki-67, CXCL10, IFN-γ, and TGFβ1 were assessed at baseline and at week 1 after treatment initiation (Figure 1, Table 2).

**Figure 2:**
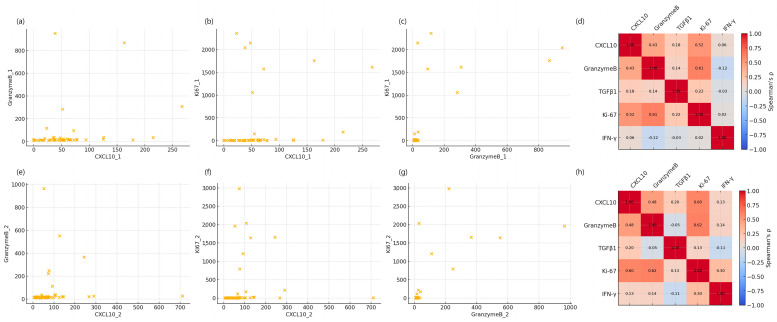
Pairwise correlations between immune markers in patients with advanced gastric cancer receiving nivolumab plus chemotherapy. (**a**) Scatter plot between CXCL10 and Granzyme B at baseline; (**b**) Scatter plot between CXCL10 and Ki67 at baseline; (**c**) Scatter plot between Granzyme B and Ki67 at baseline; (**d**) Spearman correlation matrix of immune markers at baseline; (**e**) Scatter plot between CXCL10 and Granzyme B at week 1; (**f**) Scatter plot between CXCL10 and Ki67 at week 1; (**g**) Scatter plot between Granzyme B and Ki67 at week 1; (**h**) Spearman correlation matrix of immune markers at week 1. Each dot represents an individual patient in scatter plots. Correlation coefficients (r) and *p*-values were calculated using Pearson’s correlation analysis.

Responder patients showed significant increases in Granzyme B (*p* < 0.01) and CXCL10 (*p* = 0.02), whereas these markers decreased in the non-responder group. Ki-67 and IFN-γ showed similar trends, increasing in responders and decreasing in non-responders, but without statistical significance. TGFβ1 increased in both groups, with a greater rise in non-responders (*p* = 0.16). Pairwise correlation analysis demonstrated positive associations between CXCL10, Granzyme B, and Ki-67 levels (Figure 2).

### 3.3. Prediction for Initial Immune Response

**Figure 3:**
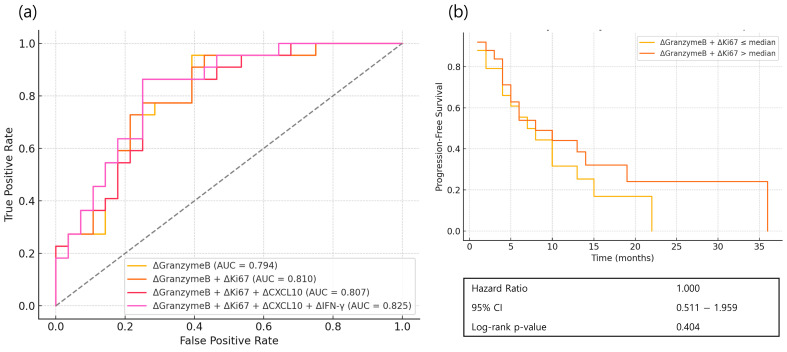
ROC curve analysis of changes (Δ, week 1—baseline) in immune markers for predicting initial treatment response and progression-free survival (PFS) in patients with advanced gastric cancer receiving nivolumab plus chemotherapy. (**a**) ROC curves of ΔGranzymeB, ΔGranzyme B + ΔKi67, ΔGranzyme B + ΔKi67 + ΔCXCL10, and ΔGranzymeB + ΔKi67 + ΔCXCL10 + ΔIFNγ for predicting initial treatment response; (**b**) PFS analysis based on the median value of ΔGranzymeB + ΔCXCL10. Statistical significance for survival analysis was determined using the log-rank test.

Receiver operating characteristic (ROC) curve analysis was performed with Granzyme B as the primary variable (Figure 3). Both ΔGranzyme B alone and the combination of ΔGranzyme B with ΔKi-67 showed effective prediction of response (AUC = 0.794 and 0.810, respectively). CXCL10 and IFN-γ may serve as supplementary indicators. These early response markers did not correspond to significant differences in PFS.

### 3.4. Prognostic Implications of Classic Immune Markers

**Figure 4:**
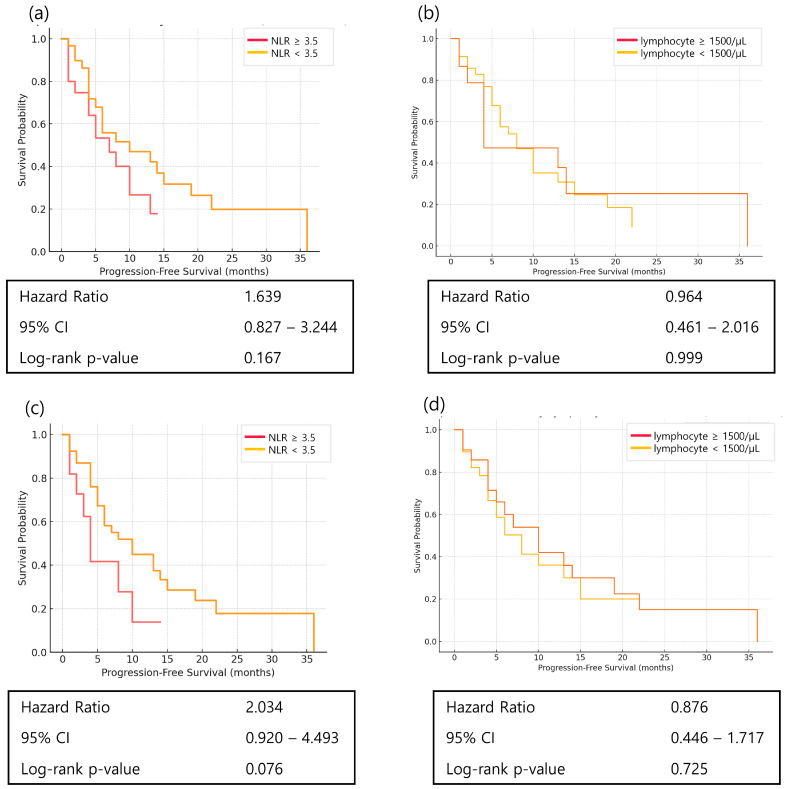
Kaplan–Meier curves of progression-free survival (PFS) according to classical immune markers in patients with advanced gastric cancer receiving nivolumab plus chemotherapy. (**a**) PFS according to baseline neutrophil-to-lymphocyte ratio (NLR) with a cut-off value of 3.5; (**b**) PFS according to baseline lymphocyte count with a cut-off value of 1500/μL; (**c**) PFS according to week 1 NLR with a cut-off value of 3.5; (**d**) PFS according to week 1 lymphocyte count with a cut-off value of 1500/μL. Statistical significance was determined using the log-rank test.

NLR and absolute lymphocyte count are established immune markers associated with prognosis in various cancers. Although NLR did not reach statistical significance in this cohort, week 1 NLR showed a trend toward prognostic relevance (HR = 2.034, *p* = 0.076) (Figure 4).

Using the 9.5-month median duration of response from the CheckMate-649 trial, patients were classified into long-term and non–long-term responders. Week 1 NLR was significantly lower in the long-term responder group (*p* = 0.045) (Table 3).

### 3.5. Prognostic Role of PD1^+^CD8^+^ T Cells and Dynamics of CD8^+^ T-Cell Subsets

**Figure 5:**
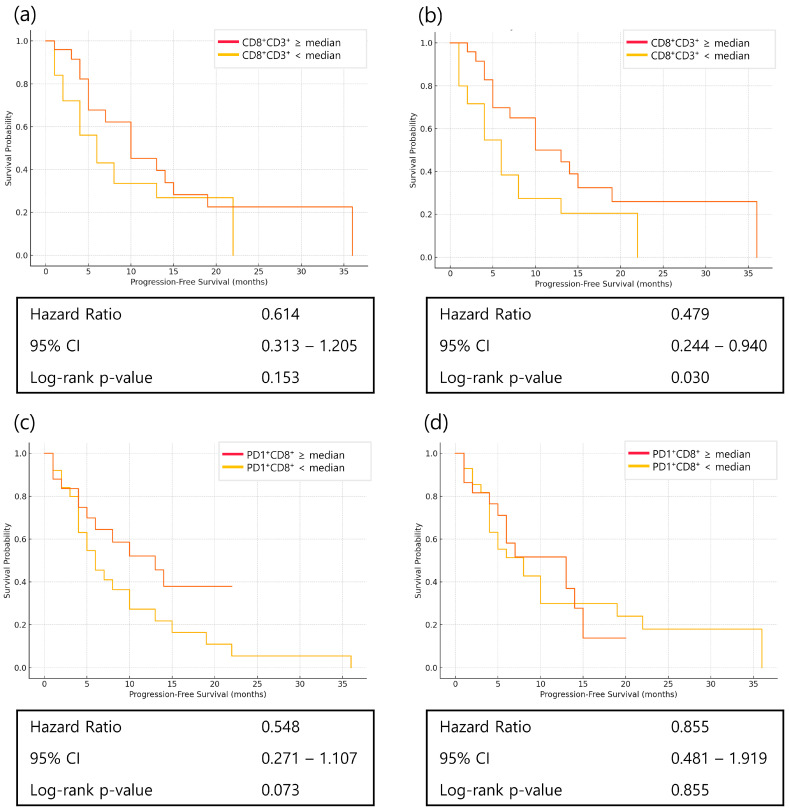
Kaplan–Meier curves of progression-free survival (PFS) according to CD8^+^ T-cell subsets at baseline and week 1 in patients with advanced gastric cancer receiving nivolumab plus chemotherapy. (**a**) PFS according to baseline CD8^+^ T-cell percentage; (**b**) PFS according to week 1 CD8^+^ T-cell percentage; (**c**) PFS according to baseline PD1^+^CD8^+^ T-cell percentage; (**d**) PFS according to week 1 PD1^+^CD8^+^ T-cell percentage. In all analyses, patients were dichotomized into high and low groups based on the median value of each marker. Statistical significance was determined using the log-rank test.

CD8^+^ T cells were analyzed by stratifying patients into high and low groups based on the median value using FACS. Patients with higher week 1 CD8^+^ T-cell proportions had significantly longer PFS (HR = 0.479, *p* = 0.030). Higher baseline PD1^+^CD8^+^ T-cell levels showed a trend toward longer PFS (HR = 0.548, *p* = 0.073) (Figure 5).

**Figure 6:**
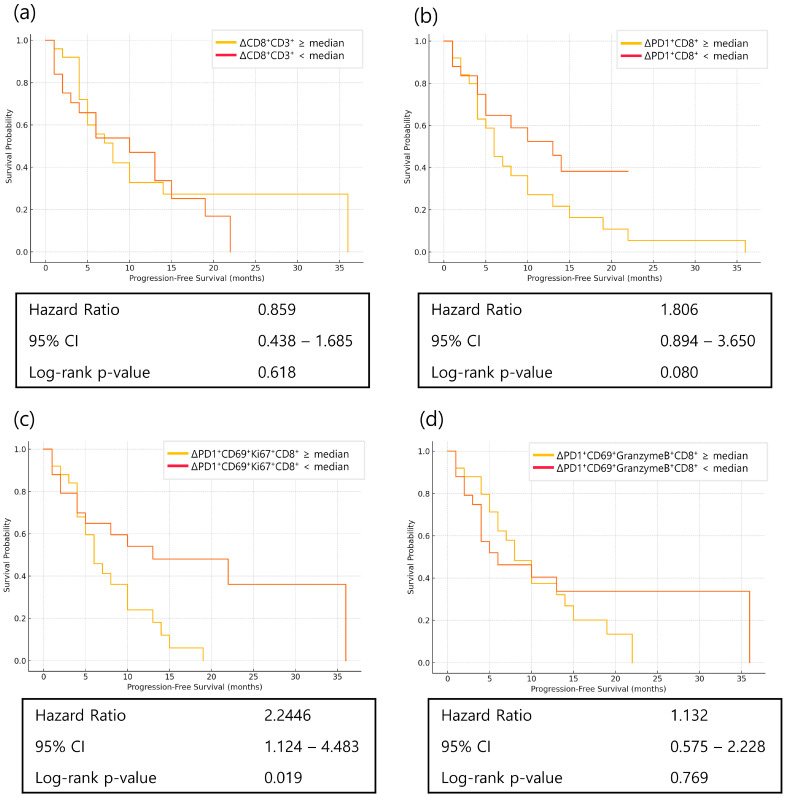
Kaplan–Meier curves of progression-free survival (PFS) according to changes (Δ, week 1—baseline) in CD8^+^ T-cell subsets in patients with advanced gastric cancer receiving nivolumab plus chemotherapy. (**a**) PFS according to ΔCD8^+^ T-cell percentage; (**b**) PFS according to ΔPD1^+^CD8^+^ T-cell percentage; (**c**) PFS according to ΔPD1^+^CD69^+^Ki67^+^CD8^+^ T-cell percentage; (**d**) PFS according to ΔPD1^+^CD69^+^GranzymeB^+^CD8^+^ T-cell percentage. In all analyses, patients were dichotomized into high and low groups based on the median value of each marker. Of note, for panels (**b**–**d**), the median Δ value was negative, indicating that most patients experienced a decrease in cell counts. Groups below the median therefore represent those with a greater reduction, which may reflect the cytotoxic effect of chemotherapy. Statistical significance was determined using the log-rank test.

Dynamic changes in CD8^+^ T-cell subsets were also examined. Both ΔPD1^+^CD8^+^ T cells and ΔPD1^+^CD69^+^Ki67^+^CD8^+^ T cells had median values below zero, indicating a decrease after treatment. Greater decreases in these subsets were associated with more favorable PFS (ΔPD1^+^CD8^+^: *p* = 0.080; ΔPD1^+^CD69^+^Ki67^+^CD8^+^: *p* = 0.019) (Figure 6).

### 3.6. Prognostic Role of Immune Checkpoint Markers in Treatment Outcomes

**Figure 7:**
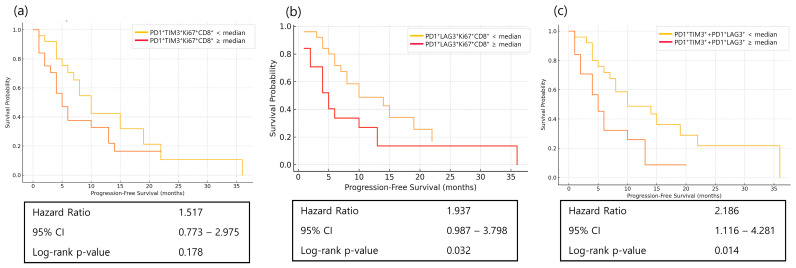
Kaplan–Meier curves of progression-free survival (PFS) according to baseline immune-checkpoint marker expression in patients with advanced gastric cancer receiving nivolumab plus chemotherapy. (**a**) PFS according to PD1^+^LAG3^+^Ki67^+^CD8^+^ T-cell percentage; (**b**) PFS according to PD1^+^TIM3^+^Ki67^+^CD8^+^ T-cell percentage; (**c**) PFS according to the combined value of PD1^+^LAG-3^+^Ki-67^+^CD8^+^ T-cell percentage and PD1^+^TIM-3^+^Ki-67^+^CD8^+^ T-cell percentage. In all analyses, patients were dichotomized into high and low groups based on the median value of each marker. Statistical significance was determined using the log-rank test.

Baseline immune checkpoint markers beyond PD1, specifically TIM3 and LAG3, were evaluated for their association with PFS. Higher baseline PD1^+^LAG3^+^Ki67^+^CD8^+^ T-cell levels were significantly associated with longer PFS (HR = 1.937, *p* = 0.032), whereas higher baseline TIM3 expression tended to be associated with longer PFS. Combining TIM3^+^ and LAG3^+^ markers resulted in a more pronounced separation of PFS curves (HR = 2.186, *p* = 0.014) (Figure 7).

### 3.7. Memory T Cells in Long-Term Responders

**Figure 8:**
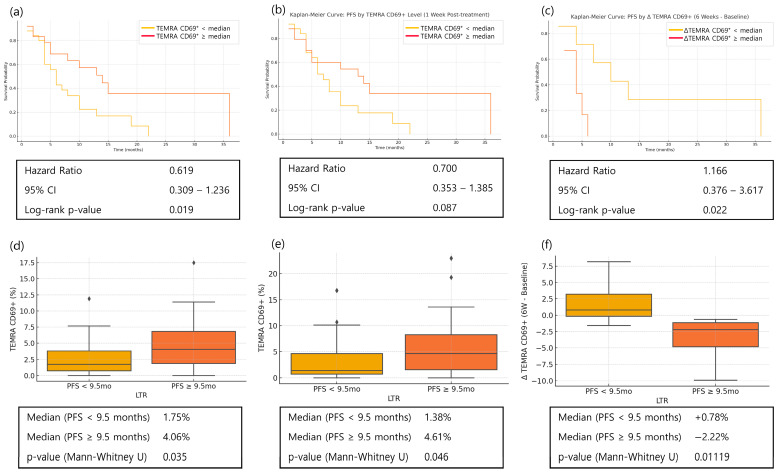
Kaplan–Meier curves of progression-free survival (PFS) and distribution of activated TEMRA (terminally differentiated effector memory CD8^+^ T cells) in long-term responders (PFS ≥ 9.5 months) and non-long-term responders (PFS < 9.5 months) among patients with advanced gastric cancer receiving nivolumab plus chemotherapy. (**a**) PFS according to baseline activated TEMRA percentage; (**b**) PFS according to week 1 activated TEMRA percentage; (**c**) PFS according to the change in activated TEMRA percentage from baseline to week 6 (Δ, median = −0.16); (**d**) Distribution of baseline activated TEMRA percentage in long-term responders vs. non-long-term responders; (**e**) Distribution of week 1 activated TEMRA percentage in long-term responders vs. non-long-term responders; (**f**) Distribution of change in activated TEMRA percentage from baseline to week 6 in long-term responders vs. non-long-term responders. For PFS analyses, patients were dichotomized into high and low groups based on the median value of each marker, and statistical significance was determined using the log-rank test. For distribution analyses, data are shown as median with interquartile range (IQR), with diamonds representing outliers, and comparisons were performed using the Mann–Whitney U test.

At baseline, higher levels of activated TEMRA cells were associated with significantly longer PFS (HR = 0.619, *p* = 0.019), and this trend persisted at week 1 (HR = 0.700, *p* = 0.087) (Figure 8). Long-term responders, defined using the 9.5-month threshold, had higher activated TEMRA levels than non–long-term responders.

Activated TEMRA cells at week 6 were analyzed in patients with available samples (*n* = 13). At this timepoint, patients with decreased activated TEMRA levels were more likely to be long-term responders (*p* = 0.011) (Figure 8).

## 4. Discussion

In this observational study, we systematically investigated dynamic immune changes in patients with advanced GC treated with nivolumab plus chemotherapy, integrating plasma biomarker profiling (ELISA) and peripheral immune cell subset analysis (flow cytometry). Our results identified distinct immunological signatures that correlated with both early treatment response and PFS, highlighting the value of serial immune monitoring in this setting.

The initial analysis focused on soluble immune markers measured by ELISA. At week 1, responders showed significant increases from baseline in plasma Granzyme B and CXCL10, both linked to cytotoxic T-cell activation and immune cell recruitment [6,11,12,13,14]. ROC curve analysis showed that ΔGranzyme B alone, and in combination with ΔKi-67, effectively predicted early clinical response. However, these early treatment-induced increases did not translate into improved PFS, underscoring a well-recognized challenge in cancer immunotherapy: initial immune activation does not necessarily ensure durable tumor control. This discrepancy may reflect the emergence of adaptive resistance mechanisms or the development of treatment-induced immune dysfunction and T-cell exhaustion [15,16].

To identify prognostic indicators more closely associated with survival, we next examined classical systemic immune markers. NLR at week 1, rather than at baseline, was more closely related to long-term outcomes, with lower week 1 NLR associated with durable benefit. This pattern echoed the ELISA results, where post-treatment changes were more informative than static baseline values [8,17]. The importance of immune dynamics prompted further investigation using cellular immune profiling by FACS.

Flow cytometric analysis revealed that patients with higher CD8^+^CD3^+^ T-cell proportions at week 1 experienced significantly longer PFS, supporting the biological plausibility that rapid immune activation after treatment initiation portends better outcomes [6]. Baseline PD1^+^CD8^+^ T-cell levels also showed a favorable trend for PFS. This association may be explained by two, non-mutually exclusive mechanisms. First, PD1^+^CD8^+^ T cells may represent antigen-experienced T cells that are functionally impaired but susceptible to restoration by PD1 blockade [15]. Second, PD1 expression may reflect recent antigen exposure and T-cell activation, thereby marking an ongoing anti-tumor immune response at baseline [8,18].

An unexpected finding was that greater decreases from baseline in PD1^+^CD8^+^ and PD1^+^Ki-67^+^CD8^+^ T cells at week 1 were linked to improved PFS. This seemingly paradoxical association may reflect the cytotoxic effects of chemotherapy not only on tumor cells but also on rapidly proliferating lymphocytes. Such treatment-induced depletion could preferentially eliminate dysfunctional or terminally exhausted T-cell populations, thereby “resetting” the immune milieu and enabling more effective anti-tumor responses—a phenomenon consistent with recent observations of immunogenic modulation by chemotherapy [19].

Baseline activated TEMRA cells were significantly associated with prolonged PFS, and long-term responders exhibited markedly higher baseline CD69^+^ TEMRA levels. While early treatment responses in our study appeared to be driven predominantly by cytotoxic activity and T-cell recruitment, these findings suggest that durable benefit may depend more on the presence of pre-existing, activation-ready memory T-cell subsets capable of sustained anti-tumor surveillance. This aligns with recent evidence that higher baseline levels of activated or antigen-experienced TEMRA cells are linked to improved long-term outcomes across several solid tumors [20,21,22]. In week 6 samples, however, we observed the opposite trend—greater decreases from baseline in activated TEMRA levels among long-term responders. Although this finding should be interpreted cautiously given the small sample size (*n* = 13), several plausible explanations exist. These include direct cytotoxic effects of chemotherapy on circulating lymphocytes, diminished antigenic stimulation as tumor burden decreases in responders, and homeostatic rebalancing of the T-cell compartment following early immune activation. Similar temporal contraction of effector populations after initial expansion has been described in other settings of effective immune responses [23,24], suggesting that this pattern may represent a physiological resolution phase rather than immune suppression. Further longitudinal studies with larger cohorts will be needed to confirm this observation.

Our analysis of immune-checkpoint markers beyond PD1 revealed that baseline LAG3 expression and combined TIM3^+^/LAG3^+^ profiles were significantly associated with improved PFS. These findings align with emerging evidence that co-expression of multiple inhibitory receptors can identify a subset of partially exhausted T cells with retained proliferative potential, suitable for functional restoration by checkpoint blockade [25,26]. This provides a robust biological rationale for dual immune checkpoint inhibition strategies, such as PD1 plus LAG-3 or PD1 plus TIM3, that are currently being evaluated across several cancers.

In melanoma, PD1 plus LAG3 blockade has yielded favorable outcomes in phase III trials [27,28]. However, in gastric cancer, results have been less promising. The phase II RELATIVITY-060 trial, which added relatlimab to nivolumab and chemotherapy, failed to improve efficacy despite selecting patients with ≥1% LAG3 expression [29], highlighting uncertainty about the optimal biomarker threshold and patient selection strategy [30]. Encouragingly, a recent phase Ib neoadjuvant trial in resectable gastroesophageal junction cancers showed acceptable safety and promising pathological response rates with nivolumab plus relatlimab in combination with chemoradiotherapy. Higher baseline PD-L1 and LAG3 expression correlated with deeper pathological responses, and ctDNA clearance was strongly associated with improved recurrence-free and overall survival [31]. Although this trial involved a different setting, the results underscore both the potential and complexity of dual checkpoint therapy in upper GI cancers. Future studies must focus on refining biomarker cutoffs and defining patient subgroups most likely to benefit from such combinations.

Taken together, our findings highlight several key points. First, early treatment-induced increases in soluble cytotoxic and chemokine markers can help identify likely responders within the first treatment week. Second, dynamic cellular immune profiling offers prognostic value beyond classical markers such as NLR. Third, baseline immune-checkpoint expression patterns and memory T-cell landscapes may inform predictions of durable benefit. Finally, certain treatment-induced decreases in specific T-cell subsets may reflect favorable chemo–immunotherapy interactions rather than immune suppression. Collectively, these observations support the integration of dynamic immune monitoring into future clinical trials to refine patient selection and enable adaptive treatment strategies, while also reinforcing the rationale for multi-checkpoint blockade and memory T cell–oriented interventions in advanced gastric cancer.

The single-center design and limited sample size, particularly for the week 6 subset, may constrain the generalizability of our findings, and the observational nature of the study precludes definitive causal inference. The week 6 analysis of activated TEMRA cells was limited to only 13 patients, not based on pre-specified selection but rather on the availability of consented patients for additional sampling, which raises the possibility of selection bias. Therefore, these results should be interpreted with great caution, and validation in larger prospective cohorts will be necessary. In addition, heterogeneity in baseline clinical characteristics and the lack of an independent validation cohort may further limit external applicability. Future investigations should aim to validate these findings in larger, multicenter cohorts and incorporate integrated tissue- and blood-based immune profiling, including ctDNA and spatial immune contexture analyses. Such approaches may not only confirm the predictive value of the identified biomarkers but also refine patient stratification for immunotherapy. Nonetheless, the serial biospecimen collection, and comprehensive immune profiling enhance the robustness and translational relevance of our conclusions.

## 5. Conclusions

In conclusion, dynamic immune profiling revealed early plasma and cellular markers that predict treatment response and prognosis in advanced GC treated with nivolumab plus chemotherapy. These findings provide a basis for biomarker-driven patient stratification and support the integration of real-time immune monitoring into future clinical trial designs.

## Figures and Tables

**Table 1:** Baseline characteristics of patients with advanced gastric cancer (*n* = 50).

Characteristic	Group	*n* (%)
Total		50 (100)
Sex	Female	22 (44.0)
	Male	28 (56.0)
Age	<65 years	38 (76.0)
	≥65 years	12 (24.0)
Histology	Adenocarcinoma	32 (64.0)
	Poorly cohesive carcinoma	18 (36.0)
Peritoneal carcinomatosis	No	19 (38.0)
	Yes	31 (62.0)
MSI-H	No	41 (82.0)
	Yes	7 (14.0)
	Unknown	2 (4.0)
HER2	Negative	21 (42.0)
	Low	29 (58.0))
	Positive	0 (0.0)
EBV	Negative	46 (92.0)
	Positive	4 (8.0)
PD-L1 CPS	<5	20 (40.0)
	≥5	28 (56.0)
	Unknown	2 (4.0)
Treatment	Nivolumab + XELOX	42 (84.0)
	Nivolumab + FOLFOX	8 (16.0)
Best response	CR or PR	28 (56.0)
	SD or PD	22 (44.0)

Abbreviations: MSI-H, microsatellite instability-high; EBV, Epstein–Barr virus; PD-L1, programmed death-ligand 1; CPS, combined positive score; XELOX, capecitabine plus oxaliplatin; FOLFOX, fluorouracil, leucovorin, and oxaliplatin.

**Table 2:** Early changes in plasma cytotoxicity and activation markers between responders and non-responders.

Marker	Responder (Mean Change)	Non-Responder (Mean Change)	*p*-Value
ΔGranzyme B	+9.81	−3.44	<0.01
ΔKi-67	+8.35	−18.13	0.17
ΔCXCL10	+48.68	−1.17	0.02
ΔIFNγ	+0.56	−0.51	0.25
ΔTGFβ1	+53.22	+268.68	0.16

Abbreviations: CXCL10, C-X-C motif chemokine ligand 10. Δ (delta) values represent week 1 minus baseline levels.

**Table 3:** Comparison of neutrophil-to-lymphocyte ratio (NLR) in long-term responders and non–long-term responders (PFS ≥ 9.5 months).

Timepoint	Group	Mean	Median	IQR (25–75%)	Max	*p*-Value
Baseline	Non-LT	3.83	3.23	1.96–4.19	20.37	0.822
	LT	3.44	2.35	1.92–4.37	10.07	
Week 1	Non-LT	2.93	2.76	1.95–3.67	8.70	0.045
	LT	2.14	1.73	1.33–2.94	5.72	

Abbreviations: LT, long-term responder; NLR, neutrophil-to-lymphocyte ratio; PFS, progression-free survival; IQR, interquartile range.

## Data Availability

The data presented in this study are available on request from the corresponding author. The data are not publicly available due to institutional restrictions.

## Abbreviations

The following abbreviations are used in this manuscript: AUC, area under the curve; CI, confidence interval; CPS, combined positive score; CR, complete response; CT, computed tomography; Δ, delta; EBV, Epstein–Barr virus; ELISA, enzyme-linked immunosorbent assay; FACS, fluorescence-activated cell sorting; FOLFOX, leucovorin, fluorouracil, and oxaliplatin; GC, gastric cancer; HR, hazard ratio; IFN-γ, interferon gamma; IJMS, International Journal of Molecular Sciences; Ki-67, antigen Ki-67 (proliferation marker); LAG3, lymphocyte-activation gene 3; MSI-H, microsatellite instability-high; NLR, neutrophil-to-lymphocyte ratio; OS, overall survival; PBMC, peripheral blood mononuclear cell; PD, progressive disease; PD1, programmed cell death protein 1; PD-L1, programmed death-ligand 1; PFS, progression-free survival; PR, partial response; RECIST, Response Evaluation Criteria in Solid Tumors; ROC, receiver operating characteristic; SD, stable disease; TGF-β1, transforming growth factor beta 1; TEMRA, terminally differentiated effector memory CD45RA^+^; TIM-3, T-cell immunoglobulin and mucin-domain containing-3; XELOX, capecitabine plus oxaliplatin.

## References

[1] Sung H., Ferlay J., Siegel R.L., Laversanne M., Soerjomataram I., Jemal A., Bray F. (2021). Global Cancer Statistics 2020: GLOBOCAN Estimates of Incidence and Mortality Worldwide for 36 Cancers in 185 Countries. CA Cancer J. Clin..

[2] Janjigian Y.Y., Shitara K., Moehler M., Garrido M., Salman P., Shen L., Wyrwicz L., Yamaguchi K., Skoczylas T., Campos Bragagnoli A. (2021). First-line nivolumab plus chemotherapy versus chemotherapy alone for advanced gastric, gastro-oesophageal junction, and oesophageal adenocarcinoma (CheckMate 649): A randomised, open-label, phase 3 trial. Lancet.

[3] Qi Y.-K., Zheng J.-S., Liu L. (2024). Mirror-image protein and peptide drug discovery through mirror-image phage display. Chem.

[4] Kim I.H., Kang S.J., Choi W., Seo A.N., Eom B.W., Kang B., Kim B.J., Min B.H., Tae C.H., Choi C.I. (2025). Korean Practice Guidelines for Gastric Cancer 2024: An Evidence-based, Multidisciplinary Approach (Update of 2022 Guideline). J. Gastric Cancer.

[5] Ghidini M., Petrillo A., Botticelli A., Trapani D., Parisi A., La Salvia A., Sajjadi E., Piciotti R., Fusco N., Khakoo S. (2021). How to Best Exploit Immunotherapeutics in Advanced Gastric Cancer: Between Biomarkers and Novel Cell-Based Approaches. J. Clin. Med..

[6] Huang A.C., Postow M.A., Orlowski R.J., Mick R., Bengsch B., Manne S., Xu W., Harmon S., Giles J.R., Wenz B. (2017). T-cell invigoration to tumour burden ratio associated with anti-PD-1 response. Nature.

[7] Kamphorst A.O., Pillai R.N., Yang S., Nasti T.H., Akondy R.S., Wieland A., Sica G.L., Yu K., Koenig L., Patel N.T. (2017). Proliferation of PD-1+ CD8 T cells in peripheral blood after PD-1-targeted therapy in lung cancer patients. Proc. Natl. Acad. Sci. USA.

[8] Shin K., Kim J., Park S.J., Kim H., Lee M.A., Kim O., Park J., Kang N., Kim I.H. (2023). Early Increase in Circulating PD-1(+)CD8(+) T Cells Predicts Favorable Survival in Patients with Advanced Gastric Cancer Receiving Chemotherapy. Cancers.

[9] Wei Y., Zhang J., Fan X., Zheng Z., Jiang X., Chen D., Lu Y., Li Y., Wang M., Hu M. (2022). Immune Profiling in Gastric Cancer Reveals the Dynamic Landscape of Immune Signature Underlying Tumor Progression. Front. Immunol..

[10] Havel J.J., Chowell D., Chan T.A. (2019). The evolving landscape of biomarkers for checkpoint inhibitor immunotherapy. Nat. Rev. Cancer.

[11] Li X., Lu M., Yuan M., Ye J., Zhang W., Xu L., Wu X., Hui B., Yang Y., Wei B. (2022). CXCL10-armed oncolytic adenovirus promotes tumor-infiltrating T-cell chemotaxis to enhance anti-PD-1 therapy. Oncoimmunology.

[12] Shi H., Chen S., Chi H. (2024). Immunometabolism of CD8(+) T cell differentiation in cancer. Trends Cancer.

[13] Tokunaga R., Zhang W., Naseem M., Puccini A., Berger M.D., Soni S., McSkane M., Baba H., Lenz H.J. (2018). CXCL9, CXCL10, CXCL11/CXCR3 axis for immune activation—A target for novel cancer therapy. Cancer Treat. Rev..

[14] Zumwalt T.J., Arnold M., Goel A., Boland C.R. (2015). Active secretion of CXCL10 and CCL5 from colorectal cancer microenvironments associates with GranzymeB+ CD8+ T-cell infiltration. Oncotarget.

[15] Baessler A., Vignali D.A.A. (2024). T Cell Exhaustion. Annu. Rev. Immunol..

[16] Zhang C., Zhang C., Wang H. (2023). Immune-checkpoint inhibitor resistance in cancer treatment: Current progress and future directions. Cancer Lett..

[17] Guo L., Li J., Wang J., Chen X., Cai C., Zhou F., Xiong A. (2024). Prognostic role of dynamic changes in inflammatory indicators in patients with non-small cell lung cancer treated with immune checkpoint inhibitors-a retrospective cohort study. Transl. Lung Cancer Res..

[18] Xu-Monette Z.Y., Zhang M., Li J., Young K.H. (2017). PD-1/PD-L1 Blockade: Have We Found the Key to Unleash the Antitumor Immune Response?. Front. Immunol..

[19] Galluzzi L., Humeau J., Buqué A., Zitvogel L., Kroemer G. (2020). Immunostimulation with chemotherapy in the era of immune checkpoint inhibitors. Nat. Rev. Clin. Oncol..

[20] Han J., Khatwani N., Searles T.G., Turk M.J., Angeles C.V. (2020). Memory CD8(+) T cell responses to cancer. Semin. Immunol..

[21] Luoma A.M., Suo S., Wang Y., Gunasti L., Porter C.B.M., Nabilsi N., Tadros J., Ferretti A.P., Liao S., Gurer C. (2022). Tissue-resident memory and circulating T cells are early responders to pre-surgical cancer immunotherapy. Cell.

[22] Virassamy B., Caramia F., Savas P., Sant S., Wang J., Christo S.N., Byrne A., Clarke K., Brown E., Teo Z.L. (2023). Intratumoral CD8(+) T cells with a tissue-resident memory phenotype mediate local immunity and immune checkpoint responses in breast cancer. Cancer Cell.

[23] Oja A.E., Piet B., Helbig C., Stark R., van der Zwan D., Blaauwgeers H., Remmerswaal E.B.M., Amsen D., Jonkers R.E., Moerland P.D. (2018). Trigger-happy resident memory CD4(+) T cells inhabit the human lungs. Mucosal Immunol..

[24] Wherry E.J., Teichgräber V., Becker T.C., Masopust D., Kaech S.M., Antia R., von Andrian U.H., Ahmed R. (2003). Lineage relationship and protective immunity of memory CD8 T cell subsets. Nat. Immunol..

[25] Andrews L.P., Cillo A.R., Karapetyan L., Kirkwood J.M., Workman C.J., Vignali D.A.A. (2022). Molecular Pathways and Mechanisms of LAG3 in Cancer Therapy. Clin. Cancer Res..

[26] Liu J., Zhang S., Hu Y., Yang Z., Li J., Liu X., Deng L., Wang Y., Zhang X., Jiang T. (2016). Targeting PD-1 and Tim-3 Pathways to Reverse CD8 T-Cell Exhaustion and Enhance Ex Vivo T-Cell Responses to Autologous Dendritic/Tumor Vaccines. J. Immunother..

[27] Long G.V., Lipson E.J., Hodi F.S., Ascierto P.A., Larkin J., Lao C., Grob J.J., Ejzykowicz F., Moshyk A., Garcia-Horton V. (2024). First-Line Nivolumab Plus Relatlimab Versus Nivolumab Plus Ipilimumab in Advanced Melanoma: An Indirect Treatment Comparison Using RELATIVITY-047 and CheckMate 067 Trial Data. J. Clin. Oncol..

[28] Tawbi H.A., Schadendorf D., Lipson E.J., Ascierto P.A., Matamala L., Castillo Gutiérrez E., Rutkowski P., Gogas H.J., Lao C.D., De Menezes J.J. (2022). Relatlimab and Nivolumab versus Nivolumab in Untreated Advanced Melanoma. N. Engl. J. Med..

[29] Hegewisch-Becker S., Mendez G., Chao J., Nemecek R., Feeney K., Van Cutsem E., Al-Batran S.E., Mansoor W., Maisey N., Pazo Cid R. (2024). First-Line Nivolumab and Relatlimab Plus Chemotherapy for Gastric or Gastroesophageal Junction Adenocarcinoma: The Phase II RELATIVITY-060 Study. J. Clin. Oncol..

[30] Seghers S., Domen A., Prenen H. (2024). Challenges and prospects of LAG-3 inhibition in advanced gastric and gastroesophageal junction cancer: Insights from the RELATIVITY-060 trial. J. Gastrointest. Oncol..

[31] Kelly R.J., Landon B.V., Zaidi A.H., Singh D., Canzoniero J.V., Balan A., Hales R.K., Voong K.R., Battafarano R.J., Jobe B.A. (2024). Neoadjuvant nivolumab or nivolumab plus LAG-3 inhibitor relatlimab in resectable esophageal/gastroesophageal junction cancer: A phase Ib trial and ctDNA analyses. Nat. Med..

